# Effect of APOE Genotype on Synaptic Proteins in Earlier Adult Life

**DOI:** 10.3233/JAD-170316

**Published:** 2017-07-29

**Authors:** Lindsey I. Sinclair, Seth Love

**Affiliations:** a School of Social and Community Medicine, University of Bristol, Oakfield House, Clifton, Bristol, UK; b School of Clinical Sciences, University of Bristol, Level 1 Learning and Research Building, Southmead Hospital, Bristol, UK

**Keywords:** Alzheimer’s disease, APOE, dementia, drebrin, genetics, postmortem tissue, PSD-95, synaptic proteins, synaptophysin

## Abstract

**Background:**

Possession of *APOE* *ɛ*4 is a strong risk factor for late-onset Alzheimer’s disease and is associated with loss of synaptic proteins in the elderly even in the absence of Alzheimer’s disease.

**Objective:**

We hypothesized that *ɛ*4 allele possession in non-demented adults aged under-75 would also be associated with alterations in the levels of synaptic proteins.

**Methods:**

We measured synaptophysin, PSD95, drebrin, SNAP-25, and septin 7 by ELISA in hippocampus and superior temporal gyrus from 103 adults aged <75 without dementia. Corresponding gene expression was measured by RT-PCR.

**Results:**

There was no evidence that *ɛ*4 affected levels of the proteins measured. Instead we found an increase in post-synaptic proteins in the hippocampi of those with an *ɛ*32 genotype. The evidence was strongest for drebrin (*p* = 0.011). There was some evidence of increased synaptic protein gene expression in *ɛ*4 carriers.

**Conclusions:**

People with an *APOE* *ɛ*32 genotype have a reduced risk of Alzheimer’s disease. It may be relevant that they have a higher level of post-synaptic proteins in the hippocampus even in earlier adulthood.

## INTRODUCTION

 Alzheimer’s disease (AD) is a devastating neurodegenerative illness which affects the lives not only of sufferers but also their caregivers. There is currently no cure. It is the sixth most common cause of death in the US and on average patients live for 4–8 years after diagnosis [1]. In addition to the classical plaques and neurofibrillary tangles, there is evidence of synaptic degeneration from an early stage of disease [2]. It is thought by some that synaptic loss is the hallmark of early AD [2].

 Although age is the strongest risk factor for developing sporadic AD, other risk factors have been identified. The strongest genetic risk factor for sporadic AD is possession of the *ɛ*4 variant of the *APOE* gene [3]. Possession of one copy of the *ɛ*4 variant increases the risk of AD three-fold and two copies increase the risk up to ten-fold [4]. The *ɛ*4 allele also decreases the age at which dementia develops but despite this many *ɛ*4 carriers do not develop dementia [5, 6]. Conversely the *ɛ*2 variant reduces the risk of AD, possibly by up to two-thirds [7–9]. Individuals with this variant have less AD neuropathology before extreme old age [10, 11]. There is evidence, predominantly from studies of middle-aged and older adults, that this variant is associated with better episodic memory performance [12]. In postmortem brain studies and in PET studies of older adults, *ɛ*4 allele possession was associated with a higher amyloid plaque load [13, 14]. In one study this effect was seen even in middle-aged individuals [15]. *ɛ*4 effects on neurofibrillary tangles are much lessconsistent [16].

The effects of *APOE* genotype in earlier adult life on the synapse are not clear. We investigated the relationship between *APOE* genotype and markers of synaptic degeneration by studying expression levels of synaptic proteins. It was not possible (at the point at which this project was planned) to study all existing synaptic proteins so we chose proteins which had been found to be affected in AD, influenced by *APOE* genotype in older adults or would provide information on an important synaptic process. For example, synaptophysin is a 313 amino acid, 38 kDa pre-synaptic vesicle-specific protein [17, 18], which can be used as a marker of synaptic content. Most studies have found a reduction in synaptophysin in AD [19–21]. Animal studies suggested that the reduction begins very early in the disease process but this finding was not replicated in human postmortem studies [22–26]. There is some evidence that *ɛ*4 carriers with AD have a greater reduction in synaptophysin than do non-*ɛ*4 carriers[19, 21].

PSD-95 is a 724 amino acid protein, which is associated with the post-synaptic membrane [17]. It is a member of the membrane-associated guanylate cyclase family [27]. Conflicting findings have been published on the changes in PSD-95 in AD. One possible explanation is that as the pre-synaptic input diminishes the size of the post-synaptic density increases initially to compensate, but the process fails with continuing neurodegeneration and PSD-95 eventually decreases. This is, however, merely one possible hypothesis to explain the observed findings. Synaptosomal associated protein (SNAP-25) is a 206 amino acid protein which is part of the SNARE complex involved in synaptic vesicle membrane docking and fusion [28]. SNAP-25 was shown to be significantly decreased in AD in some but not all studies [17, 20, 29–32]. One small study suggested that SNAP-25 is increased in *ɛ*4 carriers [33].

Drebrin, which is involved in dendritic spine morphogenesis, was shown to be reduced in dementia but relatively few studies have been performed on this protein [17, 22, 34]. Septin 7 is required for the cell cytoskeleton to be organized normally and is also involved in mitosis [17]. Although almost ubiquitous, it is expressed at particularly high levels in the CNS [35]. It is involved in dendritic spine formation, a dynamic process affected by learning [36]. Downregulation of septin 7 has been shown to reduce the complexity of the dendritic arbor [37]. Septin 7 has not been studied in AD or in relation to *APOE* genotype.

Previous work in our laboratory demonstrated reduced synaptic protein levels in older adults without dementia who possessed an *ɛ*4 allele [19]. To our knowledge, there is no published information on the effects of *ɛ*4 on synaptic proteins in younger adults. We hypothesized that synaptic proteins would be lower in non-demented adults aged 18–75 with than without an *ɛ*4 allele. The neuronal synapse is extraordinarily complex. As it was not possible to study all of the proteins present at the synapse, we made a pragmatic choice to focus on a small number proteins which had been found to be affected in AD, influenced by *APOE* genotype in older adults, or would provide information on a particular synaptic process. We measured synaptic protein concentration and gene expression in the hippocampus and superior temporal gyrus.

## METHODS

### Study cohort

Samples were obtained from the Edinburgh Sudden Death Brain and Tissue Bank (SDBTB), the South West Dementia Brain Bank (SWDBB), the Parkinson’s Disease Brain Bank, the Thomas Willis Oxford Brain Collection, and the London Neurodegenerative Diseases Brain Bank. All samples were provided for this study in compliance with the terms of Research Ethics Committee approval of the individual brain banks. A minimum age of 18 and a maximum of 75 were used for case selection. Cases with up to Braak tangle stage III pathology were included, as were those with small vessel disease and micro-infarcts some distance from the regions under investigation. Information was obtained on the presence of all of these neuropathological findings to allow later subgroup analysis. Clinical information available on the individuals under investigation was limited to the cause of their demise and any postmortem reports that were provided by the individual brain banks.

In total, samples were obtained from 103 brains; samples from both brain areas under investigation were available for 92 of these. Samples were available for RNA extraction in 190 of the 195 cases. Information on donor age, cause of death, neuropathological findings, and postmortem interval was available for all brains used in this study. The mean age at death was 56.3 y; 29 people had been less than 50 y at the time of death. Unfortunately for some individuals, the postmortem delay was longer than 72 h. Because of this, we explicitly examined between group differences in postmortem delay and included postmortem delay in the linear regressions as a co-variate.

### Cohort selection for immunohistochemistry

From the original cohort, 15 *ɛ*32 (all of those available with this genotype), 15 *ɛ*33, and 15 *ɛ*34 cases were selected for immunohistochemistry on the hippocampus. The *ɛ*33 and *ɛ*34 groups were matched to the *ɛ*32 group on age at death, gender, and postmortem delay as closely as possible. Unfortunately, paraffin-embedded hippocampal tissue was not available for all of the cases. As a result, the *ɛ*34 group numbered only 13 and the *ɛ*32 and *ɛ*33 groups each numbered 14.

### Tissue preparation

Previously-dissected frozen tissue samples from left hippocampus and superior temporal gyrus were provided by all tissue banks other than the SWDBB. Within the SWDBB, samples were dissected from slices of left cerebral hemisphere that had been frozen at –80°C at the time of initial receipt of the brains. All of the brain banks had similar procedures for sampling, fixation (usually ∼3 weeks in buffered formalin) and detailed paraffin histology on blocks of the right cerebral hemisphere, in keeping with protocols agreed by the MRC UK Brain Bank Network.

For each frozen tissue sample, 200 mg were homogenized in 1 ml chilled 1% SDS lysis buffer in a Precellys homogenizer (2×15 s at 6000×g) with 6 to 10 2.3-mm zirconia beads in a 2-ml screw cap homogenate tube. The homogenates were centrifuged at 13000×g for 15 min at 4°C. The supernatants were kept on ice and aliquoted into 25 μl aliquots and stored at –80°C until required again. Paraffin sections of hippocampus were cut at 7 μm thickness.

### Measurement of SNAP-25 by indirect ELISA

Samples were diluted in coating buffer (0.01 M sodium carbonate, 0.03 M sodium bicarbonate, pH 9.6) and 50 μl loaded per well. The recombinant protein standard (Abcam ab74529) was diluted in coating buffer, with seven 3-fold serial dilutions forming the standard curve (range = 0.003 ng/μl to 2 ng/μl). Fifty μl of standard was loaded per well. Blanks consisted of 50 μl of coating buffer. The plate was incubated at 26°C for 2 h. The plate was washed 5 times in 0.05% PBS/Tween 20 (PBST), tapped dry and non-specific protein binding blocked by incubation with 1% BSA/PBS at 26°C for 1 h with agitation. After a further 5 washes in PBST, the plate was tapped dry and the detection antibody (Santa-Cruz Biotechnology SC376713) diluted 1:3000 in 1% BSA/PBS was added for 2 h at 26°C with agitation. Following a further 5 washes with PBST, the plate was tapped dry, and horseradish peroxidase-labelled secondary antibody (Vector labs, Burlington California, USA) diluted 1:500 in PBS added for 30 min at 26°C in the dark with agitation. After a final 5 washes with PBST, the plate was tapped dry, peroxidase substrate added (R&D systems, 100 μl per well), and the plate allowed to develop for 10 min. At the end of this time, 50 μl of STOP solution was added to each well. Absorbance at 450 nm was read in a multi-detection microplate reader. In all ELISAs, absolute protein levels were determined by interpolation from the standard curve, and assays of all samples were repeated at least once in addition to being performed in duplicate on eachplate.

### Sandwich ELISA for drebrin, NSE, and synaptophysin

As previously described in detail [38], drebrin, NSE, and synaptophysin were measured by sandwich ELISAs and PSD-95 was measured by an indirect ELISA, which were designed in-house. Please refer to Table 1 for details of the antibodies used in these ELISAs. All assays used were developed using western blots to validate antibody specificity and serial recombinant protein dilutions to assess sensitivity.

**Table 1:** Antibodies used in ELISAs

Target	Provider	Antibody code	Dilution	Use
Synaptophysin	Abcam	ab 53166	1:1000	Capture antibody
Synaptophysin	Santa Cruz biotech	sc-17750	1:1000	Detection antibody
Drebrin	Abcam	ab 11068	1:3000	Capture antibody
Drebrin	Abcam	ab60932	1:3000	Detection antibody
Septin 7	Abcam	ab175229	1:500	Detection antibody
SNAP-25	Santa Cruz biotech	sc376713	1:3000	Detection antibody
PSD-95	Sigma	P246	1:3000	Detection antibody

### Measurement of Septin-7 by direct ELISA

Seven serial threefold dilutions of the recombinant protein (Cusabio, Wuhan China, CSB-EP620952HU), and samples, all diluted 1:40 in 1x coating buffer, were loaded in duplicate in clear 96-well microplates prior to incubation at 4°C overnight. The range of the standard was 0.025 to 18.18 ng/μl. The rabbit monoclonal antibody (Abcam ab175229) used for detection was biotinylated with Lightning Link modifier kit (Innova Biosciences, Cambridge UK), incubated overnight at 4°C and then quenched prior to use. It could then be stored at 4°C for up to a week until required.

After overnight incubation, the plate was washed with PBST, tapped dry, and non-specific protein binding was blocked by incubation with 5% milk/PBS at room temperature for 90 min at 26°C with agitation. After further washes in PBST, the plate was tapped dry. The biotinylated detection antibody, diluted 1:500 in 1% BSA/PBS, was added at room temperature for 2 h at 26°C with agitation. Bound antibody was measured as above.

### Postmortem protein stability

NSE mRNA and protein levels were previously shown not to be affected by postmortem delay for at least 72 h [39]. Synaptophysin, SNAP-25, and drebrin protein concentrations were also previously shown to be stable for up to 72 h postmortem [19, 38, 40, 41]. PSD-95 was previously shown to decrease slightly after 72 h at room temperature but not when stored at 4°C [38].

Postmortem stability of septin-7 has not previously been assessed. Postmortem delay was simulated by dissecting a piece of tissue from the anterior frontal cortex of two cases with AD and two control brains, all with a short postmortem delay (cases 4 and 5 h, controls 5.5 and 6 h). The tissue was subdivided into 10 small pieces of equal weight. One piece was frozen immediately at –80°C. The others were stored at 4°C for 24, 48, or 72 h, or at room temperature for 6, 12, 18, 24, 48, or 72 h. At the end of the simulated postmortem delay all of the samples were homogenized in SDS lysis buffer and frozen at –80°C until required. As shown in Supplementary Figure 2, septin 7 was stable for up to 72 h under simulated postmortem conditions.

### Measurement of mRNA

As described previously, mRNA was extracted from frozen brain tissue [40] and RNA concentration determined by ribogreen quantification (Invitrogen kit) before conversion to cDNA using a high-capacity reverse transcription kit (Applied Biosystems, Foster City California, USA) [39]. Real-time PCR was carried out using a Viia7 real-time PCR system. Taqman gene expression assays (Applied Biosystems) which crossed exons were used for MAP2 (Hs00258900_m1), NSE (Hs00157360_m1), GAPDH (Hs02758991_g1), SEPT7 (Hs00987502_g1), SYP (Hs00300531_m1), PSD-95 (Hs00176354), DBN1 (Hs00365623_m1), and SNAP-25 (Hs00938962_m1). Gene expression samples were incubated with Taqman advanced fast mastermix and 10 ng of cDNA (total volume of 20 μl) at 50°C for 2 min, 95°C for 20 s and 40 cycles of 95°C for 1 s, then 60°C for 20 s. Samples were analyzed in triplicate.

### Choice of reference genes

It is usual when performing gene expression studies to determine the expression of the gene of interest relative to that of a calibrator or reference gene, often a so-called housekeeping gene and sometimes a gene that is chosen because it has the same restricted cell-type specific pattern of expression as the gene of interest. This can help to reduce other sources of variation, for example in overall RNA quantity or (to some extent) quality, or in the relative number of different cell types. *GAPDH* is a commonly used reference gene as it is ubiquitously expressed at high levels. It is less suitable for studies such as this one precisely because it is ubiquitously expressed, as discussed in detail in Palmer et al. [42]. In keeping with standard practice in our laboratory, for calibration we therefore also used neuron-specific reference genes, the expression of which within neurons is not directly affected by AD: microtubule associated protein 2 (*MAP2*) and neuron specific enolase (*ENO2*) [40].

### APOE genotyping

All ELISAs and RT-PCR assays were carried out blind to *APOE* genotype. Initial genotyping of all study participants for *APOE* was undertaken by integrated single-label liquid phase assay. Full details of this method were published previously [43].

When the initial genotyping was performed there appeared to be an excess of *ɛ*4 alleles (specifically, a large number of *ɛ*44 genotypes) and the genotypes were not in Hardy-Weinberg equilibrium. As the most likely cause of this was genotyping error all samples thought to have a genotype containing an *ɛ*4 allele were re-typed by Cfo1 restriction digestion— a method based on Wenham et al. [44]. This method is the standard *APOE* typing method used in the South West Dementia Brain Bank but is not well suited to high-throughput studies. Typing was performed by examining the digestion products under UV light after electrophoresis in a 3.5% agarose gel with ethidium bromide.

### Immunoperoxidase labelling of paraffin sections

The paraffin sections were incubated at 60°C overnight, then de-waxed in clearene (2×5 min) and dehydrated in 100% alcohol (Fisher scientific, 2×3 min). Endogenous peroxidase activity was blocked by incubation in 3% H_2_O_2_ in 100% methanol for 55 min before the sections were washed in running water for 10 min. Antigen retrieval was performed by boiling the sections in citric acid. The sections were heated until boiling, then cooled for 5 min before being heated again and boiled for 1 min. They were then cooled for 20 min before being washed in running water. Non-specific antibody binding was blocked with 20% normal horse serum (Vector labs) for 2 h. The sections were washed 2×3 min in PBS before being incubated with the primary antibody overnight (PSD-95, Sigma Aldrich P246; MAP2, Sigma Aldrich M4403; drebrin, Abcam ab60932).

The following day the sections were washed in PBS for 2×3 min, incubated with the universal secondary antibody (Vector labs) for 20 min, washed again in PBS and incubated with Vecta Elite ABC complex (Vector labs) for 20 min. After further washes in PBS the sections were incubated with DAB (Vector labs) for 10 min before being washed in running water for 10 min. They were then placed in 0.16 M copper sulphate for 4 min before a further wash in running water for 5 min. They were counterstained in Harris’s hematoxylin for 20 s and washed again in running water for 15 min prior to dehydration, clearing, and mounting.

The field fraction immunopositive for each antigen was assessed by examining up to 20 random fields of the cornu ammonis (CA1, 2, and 3 subfields) under a 20x objective. The software package Image Pro Plus (Media Cybernetics, MD, USA) was used to select the fields at random in the pre-defined area and for image capture. For a small number of slides, the area of included cornu ammonis was too small to accommodate 20 non-overlapping fields but at least 10 were captured in all cases.

### Double immunofluorescent labelling of paraffin sections

These sections were dewaxed in the same manner as the immunohistochemistry slides. After de-waxing the sections were washed in running water for 10 min before citric acid antigen retrieval as above. The sections were blocked in 10% normal horse serum/0.1% triton/PBS for 2.5 h (Vector labs, Sigma Aldrich) before being incubated with the primary antibodies diluted in 10% normal horse serum/0.1% triton/PBS overnight (MAP2, Abcam ab5392, drebrin Abcam ab11068 at 4°C. After 3×3 min washes in PBS the sections were incubated for 2 h with Alexa fluor-conjugated secondary antibodies (Invitrogen) diluted 1:100 in 10% normal horse serum/0.1% triton/PBS. Following this the sections were washed 3×3 min in PBS prior to incubation in cupric sulphate (0.05 M ammonium acetate, 0.44 mM copper II sulphate, pH 5.0) for 45 min. After quenching for 3×3 min in dH_2_O the sections were placed in 3% Sudan black in 70% ethanol (Sigma Aldrich) for 6 min. The sections were briefly dipped in 70% ethanol, then in dH_2_O, mounted in vectashield (Vector labs) and sealed with clear nail varnish. Representative images were taken of the stratum radiatum of CA1.

### Statistical analysis

All RT-PCR and protein assays were performed blind to *APOE* genotype. Parametric statistical tests were used where possible. If variables were not normally distributed, logarithmic transformation was used to obtain a normal distribution, if possible. For normally distributed variables linear regression or ANOVA was used. For variables that were not normally distributed, even after logarithmic transformation, Kruskal Wallis test and Dunn’s *post hoc* test were used.

Pearson’s correlation was used to assess the effect of postmortem delay. Due to the difficulties in recruitment to this study, some tissue samples with a long postmortem delay (>72 h) were included. As protein stability after 72 h had not been assessed experimentally we made an *a priori* decision to conduct sensitivity testing by re-running the analyses after exclusion of samples with a postmortem delay >72 h (see Supplementary Material). By using these two methods, we could be more confident that any findings were not an artefact of variation between groups in postmortem delay. In addition, where postmortem delay was shown to affect protein level, we used linear regression with postmortem delay included as a variable. If the residuals were not normally distributed (and thus the assumptions of linear regression violated), the variable was log transformed and the distribution of the residuals re-checked. A threshold for *p* values of 0.05 was used throughout.

RT-PCR results were analyzed by the 2^-*ΔΔ*Ct^ method [45]. This yields relative quantification rather than absolute values for expression. Expression is normalized to reference (calibrator) genes. This controls for errors due to loading of differing amounts of cDNA, operator handling, and differing intra-assay variability. It is also suitable for studying low expression levels [45]. It is considered to be the best method for comparing expression between groups and by inclusion of appropriate calibrators can be used to quantify expression restricted to a single type of cell within a mixed population of cell types.

To maximize study power, the data were initially analyzed as *ɛ*4 versus non-*ɛ*4. To explore the findings further, follow-up analysis used most of the *APOE* genotype groups but was limited by low study power. We made an *a priori* decision to exclude individuals with an *ɛ*42 genotype from analyses as this group mixes the highest and lowest risk alleles. This is standard practice in *APOE* research [46]. As each individual variable is a crude proxy measure of pre- or post-synaptic density and has measurement error, a summary Z score was also used. This was calculated by normalizing each individual variable (x) to the mean of that variable (μ), i.e., calculating (x-μ)/*σ* where *σ* is the standard deviation, and then summing the Z scores of individual variables to generate a pre- and post-synaptic Z score[19, 47].

Power calculations based on previous published work suggested a larger sample than we were able to acquire from UK brain banks. For synaptic proteins, taking synaptophysin as an example, the sample size required to find a 5 OD/mg difference between the 2 groups with 80% power and an α of 0.05 would be 63 in each group. It was not possible to perform a formal power calculation for the RT-PCR study [19].

### Assay reliability

Although most of the assays generated reproducible values, the SNAP-25 and drebrin assays had lower reproducibility. The average measure intra-class correlation co-efficients (ICCs) for synaptophysin, PSD-95, and septin 7 were 0.831, 0.846, and 0.605 respectively, indicating excellent reproducibility. The ICC for the drebrin assay was 0.174, indicating low agreement between measurements on different plates. The ICC for the SNAP-25 assay was –0.103 for single measures and –0.389 for average measures, indicating a low level of reproducibility.

## RESULTS

As shown in Table 2, this was a relatively middle-aged cohort with a mean age at death of 56.1 y (SD 13.3 y). It included tissue from 56 individuals aged <50 at death. *APOE* genotypes were available for all samples. Following our re-typing by the Cfo1 method the genotypes were in Hardy-Weinberg equilibrium (*χ*^2^ = 2.007, *p* = 0.571). There was no evidence of a between-genotype group difference in age, gender, postmortem delay, Braak stage, or cause of death (see Table 1).

In total, tissue was received from 103 individuals. After exclusion of those with an *ɛ*42 genotype, we had 95 samples of hippocampus and 100 of superior temporal gyrus. For a small number of individuals, tissue was not available for RNA extraction; in total 190 samples were available for RNA extraction. Braak staging was performed by the modified method of Braak et al. [48]. Because some of the sections of temporal lobe from other brain banks did not include the transentorhinal region and a few contained only part of the full coronal extent of the hippocampus, it is possible that some of the brains assigned a Braak stage 0 were actually Braak stage I, and a few assigned a Braak stage I were actually Braak stage II. However, we could be confident that individuals with AD were not included in our sample, as individuals with Braak stages I-II have a low probability of AD [49].

### Synaptic protein measurements

There was no evidence of a per-*APOE* genotype difference in NSE, synaptophysin, or SNAP-25 in either brain region (see Tables 2 & 3). The initial ANOVA was strongly suggestive of a difference in drebrin in the hippocampus between the genotype groups. Subsequent linear regression showed that this was due to an increase in drebrin in the *ɛ*32 group (β= 0.632, 95% CI 0.285 to 0.943, *p* < 0.001). This finding was unchanged when age-at-death and postmortem delay were included as co-variates in a sensitivity analysis (see Supplementary Tables 3 and 4). As shown in Supplementary Figure 1, it was also not associated with Braak tangle stage. Although the same trend was seen for PSD95 and septin 7 in the hippocampus, the increase in these proteins was not statistically significant. The summary post-synaptic Z score supported the finding of a rise in post-synaptic proteins in the hippocampus in the *ɛ*32 group. As the data were not normally distributed, Kruskal Wallis test and Dunn’s *post hoc* test were used. The *ɛ*32 group differed significantly from the *ɛ*33 (*p* = 0.0007) and *ɛ*34 (*p* = 0.0019) groups, and there was a trend toward a difference from the *ɛ*44 group (*p* = 0.0865).

**Table 2:** Demographics and information on key variables for the brain bank cohort

	*ɛ*33		*ɛ*34		*ɛ*44		*ɛ*32		*ɛ*22		Statistical group evidence for a
	(*n* = 57)		(*n* = 22)		(*n* = 5)		(*n* = 15)		(*n* = 1)		between difference
	mean	SD	mean	SD	mean	SD	mean	SD	mean	SD	(*χ*^2^, F-test, Kruskal-Wallis or ANOVA)
Age at death (y)	56.5	13.6	57.8	12.0	40.2	13.0	61.5	11.9	48		Kruskal Wallis
											*χ*^2^ = 8.746, *p* = 0.068
Gender											*χ*^2^ = 1.835 *p* = 0.766
Female	12		4		0		2		0	
Male	46		18		5		13		1	
Cause of death											*χ*^2^ = 32.340
CVD	26		15		3		3		1		*p* = 0.643
Cancer	10		3		0		6		0	
Respiratory	7		1		0		1		0	
Trauma	2		0		1		3		0	
Postmortem delay (h)	51.2	25.6	51.8	17.7	62.0	15.2	47.3	26.6	95		Kruskal Wallis
											*χ*^2^ = 4.752, *p* = 0.314
Braak stage											Kruskal Wallis
0	14		4				6				*χ*^2^ = 0.003
I	9		4				1				*p* = 0.998
II	6		2				3			
III	2		0				1			
Neuropathology findings											*χ*^2^ = 18.130
Normal	38		13		4		12		1		*p* = 0.796
Mild age-related changes	5		2		0		0		0
Small vessel disease	10		7		1		0		0
Other	4		0		0		3		0
STG NSE (ng/μl)	2.809	1.447	2.775	1.699	2.282	1.544	3.227	1.467	2.789	*p* = 0.875
Hippocampal NSE (ng/μl)	2.774	1.516	2.147	1.528	3.720	2.940	2.558	1.123	1.386	*p* = 0.180

#### Gene expression

Initial analysis of *ɛ*4 versus non-*ɛ*4 revealed a significant increase in *ɛ*4 carriers in the expression of *DLG4* (the gene for PSD-95) compared to all 3 reference genes (see Figs. 2 and 6, Supplementary Tables 23 and 25) in the hippocampus. This was not seen in the analysis by individual genotypes, possibly due to a reduction in power, although the result for *MAP2* was suggestive of a possible effect (see Supplementary Table 3). We detected no other hippocampal effects in *ɛ*4 versus non-*ɛ*4 analysis.

**Fig. 1:**
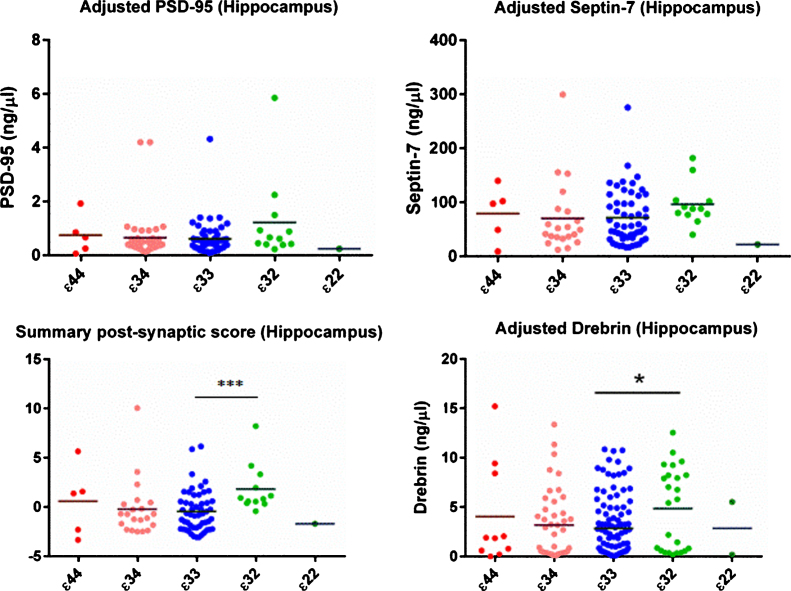
Post-synaptic protein levels in the hippocampus. All protein concentrations were adjusted for total protein and neuronal content. As shown the evidence was strongest for an increase in drebrin in the *ɛ*32 group, although the same pattern was seen for all three post-synaptic proteins. The summary/Z score provided further statistical evidence of a between-group difference.

**Fig. 2:**
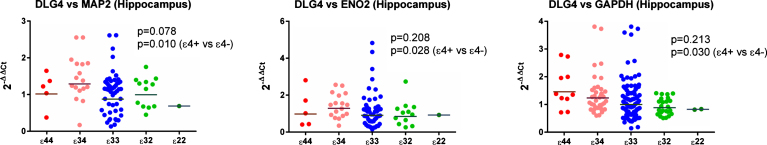
Gene expression of DLG4 (gene for PSD95) in the hippocampus. As shown there was evidence of a between-group difference with respect to all three reference genes.

**Fig. 3:**
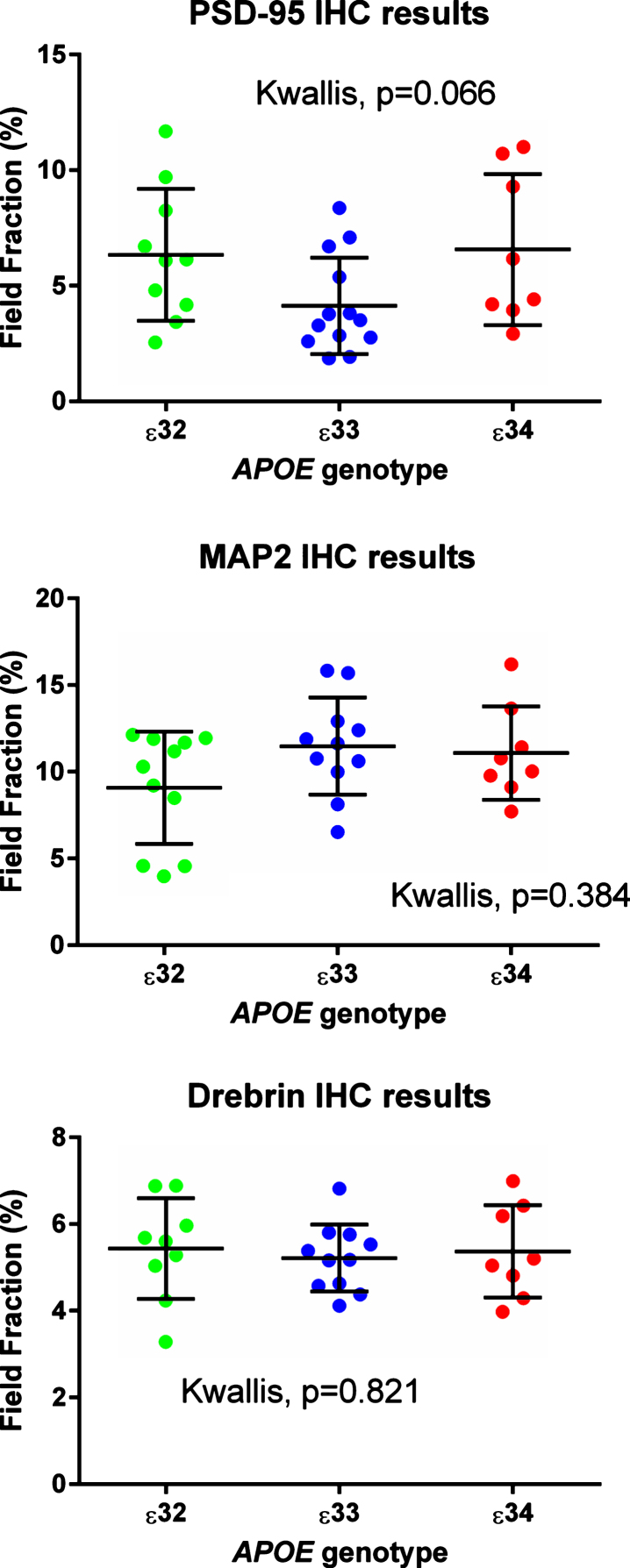
Results from the immunohistochemical (IHC) field fraction study. Areas under investigation were CA1, 2, and 3.

**Fig. 4:**
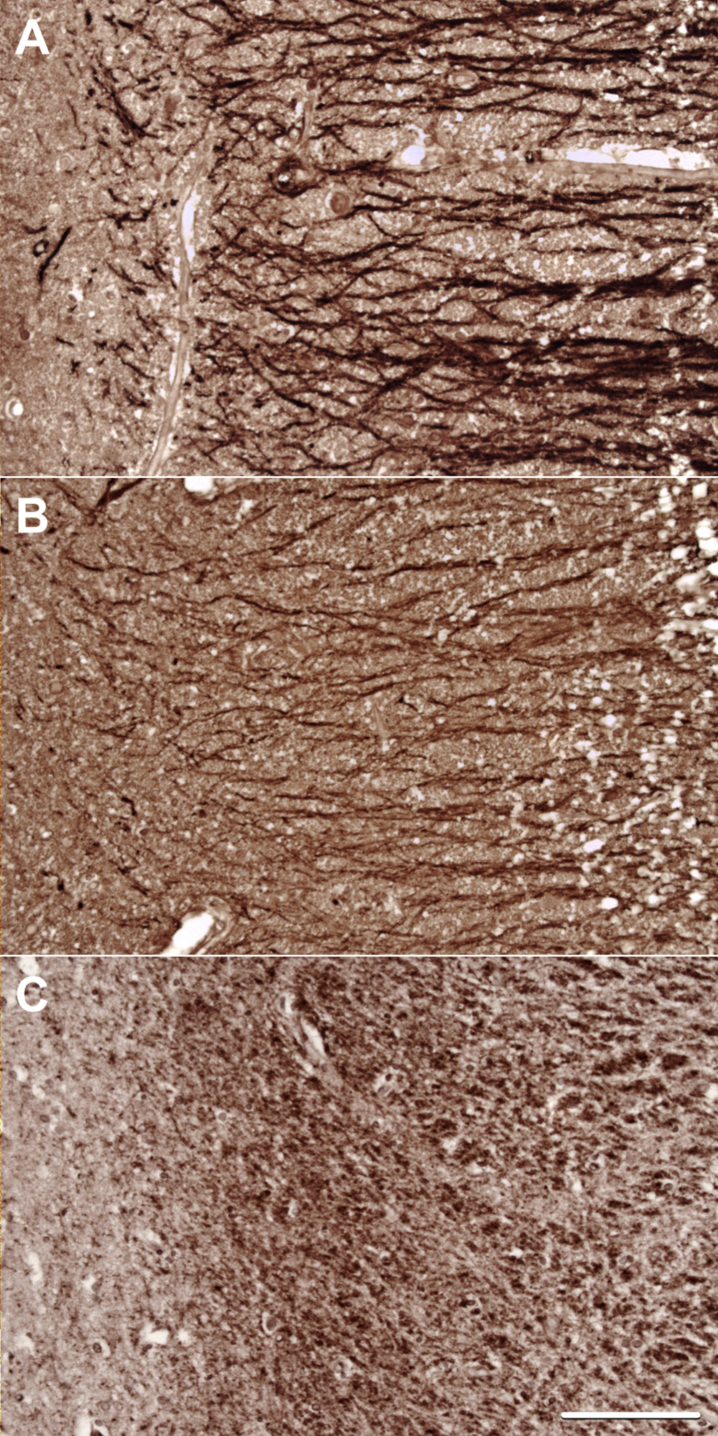
Representative images of staining for (A) MAP2, (B) Drebrin, and (C) PSD-95 in the stratum radiatum of CA1.

**Fig. 5:**
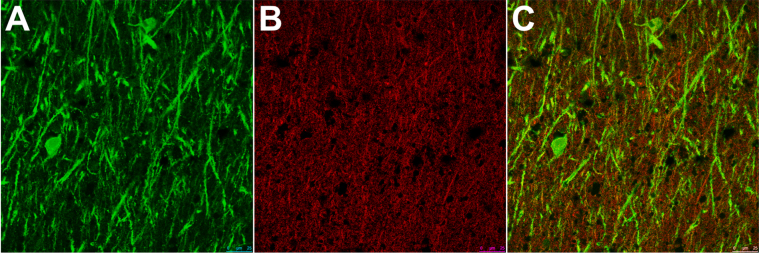
Representative double labelled immunofluorescence staining of (A) MAP2, (B) drebrin, and (C) combined in the stratum radiatum of CA1. As can be seen, the staining for drebrin is very punctate but does have some overlap with MAP.

**Fig.6:**
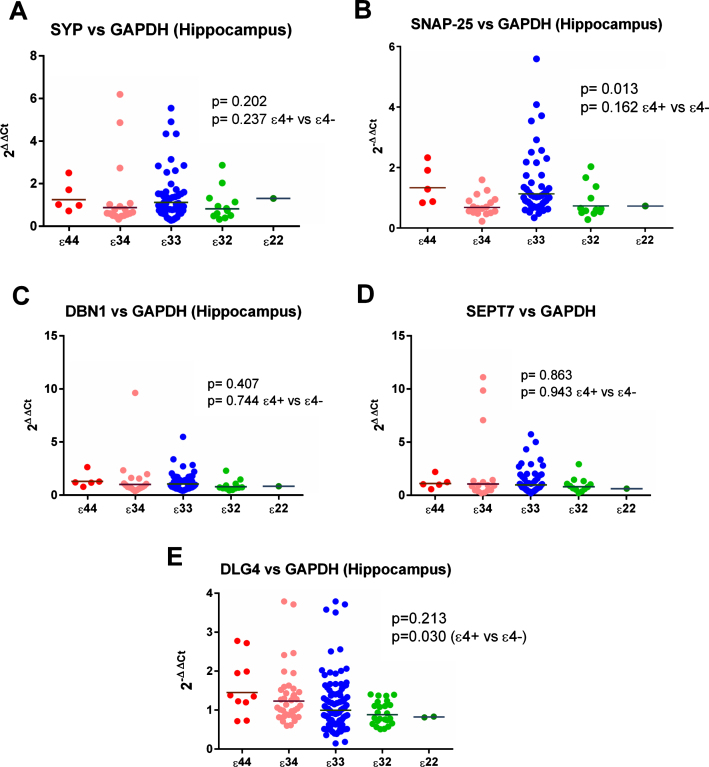
Results from the gene expression study in the hippocampus with GAPDH used as the reference gene. As shown in (E), expression of DLG4 (the gene for PSD-95) appeared to be increased in *ɛ*4 carriers.

**Fig.7:**
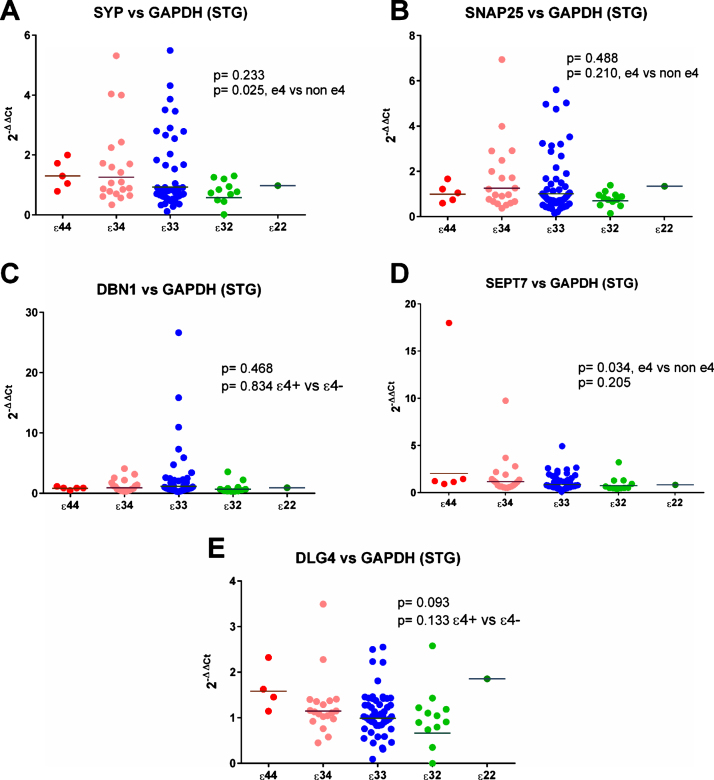
Results from the gene expression study in the superior temporal gyrus (STG) with GAPDH used as the reference gene. As can be seen in (A) and (D) there was a suggestion that expression of SYP and SEPT7 was increased in *ɛ*4 carriers but there was no evidence of any other differences between the APOE genotype groups.

*SYP* (synaptophysin) expression was increased significantly relative to *ENO2* and *GAPDH* in the superior temporal gyrus in *ɛ*4 carriers (see Fig. 7), and non-significantly relative to *MAP2* (see Supplementary Tables 24 and 26). Analysis by individual genotype (see Supplementary Table 4) did not reveal strong evidence of a per-genotype difference in gene expression.

#### Immunohistochemistry

Antibodies to MAP2, drebrin, and PSD-95 labelled cornu ammonis dendrites, particularly those in the stratum radiatum (Fig. 4), and neuronal perikarya. Whereas the immunolabeling of dendrites for MAP2 and drebrin appeared continuous, the pattern of labelling of PSD-95 was punctate or granular. As shown in Fig. 5, there was overlap in the distribution of MAP2 and drebrin. As shown in Supplementary Table 27 and Fig. 3, there was no clear evidence of a difference in the field fraction immunopositive for either drebrin or MAP2 in the hippocampus. There was a trend toward a lower PSD-95 field fraction in the *ɛ*33 group (*p* = 0.066). Unfortunately, the limited availability of cases with a sufficient amount of residual hippocampus in the paraffin blocks for immunohistochemical quantification of these antigens reduced the power of this part of the study.

## DISCUSSION

In this relatively large series of normal brains from adults aged between 18 and 75 y, we have found evidence of an increase in post-synaptic proteins in the hippocampus of those with an *APOE* *ɛ*32 genotype. The evidence was strongest for drebrin, but as shown in Fig. 1, the same direction of effect was seen for all three post-synaptic proteins measured. The post-synaptic Z score for the hippocampus provided further evidence in support of an increase in post-synaptic proteins in the *ɛ*32 genotype group. Contrary to our hypothesis, we did not find a difference in synaptic protein levels (as measured by ELISA) in those with an *ɛ*4 *APOE* allele compared to those without, and there was no relationship between levels of pre-synaptic proteins and *APOE* genotype in either the hippocampus or the superior temporal gyrus. The results are summarized in Table 3.

**Table 3:** Summary of the study findings

Protein	Brain Area	ELISA	Immunohistochemistry	Gene expression (RT-PCR)
Synaptophysin	Hippocampus	↔*	N/A	↔
	Superior Temporal Gyrus	↔	N/A	Increased in *ɛ*4 carriers compared to 2 reference genes
SNAP-25	Hippocampus	↔	N/A	Decreased in *ɛ*2 carriers compared to 2 reference genes
	Superior Temporal Gyrus	↔	N/A	↔
Drebrin	Hippocampus	Small increase in *ɛ*32 group	↔	↔
	Superior Temporal Gyrus	↔	N/A	↔
PSD-95	Hippocampus	Trend towards an increase in *ɛ*32 group	No strong evidence of a per genotype difference	Increased in *ɛ*4 carriers compared to all 3 reference genes
	Superior Temporal Gyrus	↔	N/A	↔
Septin 7	Hippocampus	↔	N/A	↔
	Superior Temporal Gyrus	↔	N/A	↔

*↔, no evidence of a per-genotype difference.

Some animal studies have suggested that the concentration of synaptophysin in the hippocampus starts to decrease very early in the development of AD. In one study, mice as young as 4 months with the human *APOE* *ɛ*4 gene exhibited a reduction in synaptophysin [24], although in another study no difference could be seen before 12 months (equivalent of human old age) [26]. Human studies have provided some evidence that synaptophysin may increase at an early stage in AD before decreasing as the dementia becomes more severe [23]. The lack of effect of the *ɛ*4 allele in the present study may be because our cohort was too young or may be due to a genuine null effect.

Although drebrin has been shown to be reduced in AD, it has not previously been studied in relation to *APOE* genotype in the absence of AD [22, 34]. PSD-95 was reduced in older non-demented controls in one study, but the relationship of this to AD is unclear [19]. Two recent studies suggested that PSD-95 declines relatively late in the AD disease process and that it is redistributed from apical dendrites to the nerve cell body as Aβ and tau pathology worsen [50, 51]. If the reduction is indeed later in the disease process, then this may explain our null finding. Previous research has not established a clear relationship between SNAP-25, *APOE*, and AD. Again contrary to our hypothesis, we did not find reduced transcript levels of synaptic proteins in *ɛ*4 carriers. Instead there was increased *DLG4* gene expression in the hippocampi of *ɛ*4 carriers in relation to all three reference genes. In the superior temporal gyrus, there was evidence of increased expression of the genes for three synaptic proteins in *ɛ*4 carriers. This evidence was most robust for the *SYP* gene although not in relation to *MAP2*. The dissociation between synaptic protein and transcript levels has a number of possible explanations. Protein tends to be more stable postmortem than mRNA, although this too varies depending on the protein. In the present study, we measured proteins that we had shown to be relatively stable under postmortem conditions. Fleige et al. [52] showed that RNA integrity (as indicated by the RIN value) influenced the Ct of RT-PCR but that this effect was reduced (although not eradicated) by normalization to a reference gene. Fleige et al. also commented that RT-PCR amplicons of <250 bp generate results that are to some extent independent of the quality of the initial RNA [53] and they recommended using amplicons no longer than 200 bp to obtain reliable RT-PCR results. An independent group replicated these findings [54]. The amplicons used in this study were all≤111 bp and mostly <100 bp. Several previous authors have found weak or inconsistent correlation between mRNA and protein levels [55–58].

Carriage of an *ɛ*2 allele is known to reduce the risk of an individual’s developing AD, possibly by up to two-thirds [7–9]. Some, but not all, studies have suggested that this protection may lessen in extreme old age as other factors, such as vascular disease, become more important than neurofibrillary tangles and amyloid plaques in the development of dementia. There is some evidence that below the age of 80, *ɛ*2 carriers have less AD neuropathology but that after this age they have as much as those with *ɛ*4 alleles [10, 11]. In the (relatively small) study by Berlau et al., *ɛ*2 carriers had significantly more AD neuropathology than the reference *ɛ*33 group but a reduced likelihood of dementia (OR of 0.3) [10]. In a follow-up study, non-demented *ɛ*2 carriers had smaller, denser amyloid plaques than *ɛ*33 homozygotes or *ɛ*4 carriers [59]. The authors wondered whether the E2 protein sequestered Aβ into plaques more effectively than other isoforms of apolipoprotein E, reducing the level of toxic oligomers. They also speculated as to whether some other unknown compensatory mechanism is present in those with an *ɛ*2 allele [10, 59]. Recent research by Chung et al. which showed that the E2 isoform of ApoE increased synaptic phagocytosis by astrocytes (with possible evidence of fewer senescent synapses) may explain some of the findings [60].

The E2 protein has been shown in animals to increase early dendrite development, to bind Aβ more strongly and to be more resistant than the other isoforms to degradation [61–63]. Several studies have demonstrated that E2 promotes dendrite formation or regeneration. In a small study in mice, possession of *ɛ*2 protected against dendritic spine loss in the hippocampus of mice transgenic for APP [64]. An interesting transgenic mouse experiment (human *APOE* gene, mouse promoter), in which *ɛ*2 was, unfortunately, studied only in combination with *ɛ*4, found that *ɛ*2 reversed synaptic deficits detected by electrophysiology in brain slices but did not fully reverse the reduction in dendritic arbors at 7 months of age [65]. A further *in vitro* study, in which wild type mouse neurons were co-cultured with transgenic mice astrocytes, found that compared to E3 and E4 astrocytes, E2 astrocytes accelerated the development of dendritic spines [66]. As mature dendritic spines contain NMDA receptors and PSD-95 is an NMDA scaffold protein, this may explain the observed changes in both drebrin and PSD-95 [66]. In contrast, a study of neurite outgrowth in cultured mouse olfactory epithelial cells found that recombinant E2 promoted neurite outgrowth less than E3 but more than E4 [67]. A previous study by our group found a 17% increase in PSD-95 in the superior temporal cortex of older adults without dementia who had at least one *ɛ*2 allele [19]. A small transgenic mouse study, which used western blots to measure PSD-95 and drebrin, also suggested (but did not prove) that drebrin may be increased in *ɛ*2 carriers [68].

Most studies in the field of AD have looked at those who either have pathological abnormalities or who are elderly and at high risk. We set out to study the effects of *APOE* on synaptic proteins in early to mid-adult life. Contrary to our hypothesis that *ɛ*4 carriers would have lower levels of synaptic proteins and lower expression of the relevant genes we did not find any differences in this group at elevated genetic risk of AD. Instead we found possibly protective differences in carriers of the *ɛ*2 allele, which reduces the risk of AD.

## ETHICAL APPROVAL

Ethical approval for this research was provided by the generic ethical approval for the participating brain banks for the approval of peer reviewed research. The details of these are as follows; SWDBB (08/H0106/28 + 5, Central Bristol REC); Thomas Willis Collection Oxford (07/0606/85); Parkinson’s Disease Brain Bank London (08/MRE09/31 + 5, REC for Wales); Sudden Death Brain and Tissue Bank (SDBTB) Edinburgh (/ES/0022, EoSREC 1); London Neurodegenerative Diseases Brain Bank (08/MRE09/38 + 5, REC for Wales).

## Supplementary Material

Supplementary Material
