# Supplementary material for: Effect of APOE Genotype on Synaptic Proteins in Earlier Adult Life
Source: J Alzheimers Dis. 2017 Jul 29;59(3):1123–37. doi: 10.3233/JAD-170316 (PMC5545905; doi:10.3233/JAD-170316)
Supplement: Supplementary Material [file jad-59-jad170316-s001.docx]

**Supplementary Information**

Contents

[ELISA results 2](#_Toc485120463)

[The effect of postmortem delay 3](#_Toc485120464)

[The effect of Braak Stage 4](#_Toc485120465)

[Drebrin sensitivity analysis 6](#_Toc485120466)

[Synaptophysin Sensitivity Analysis 7](#_Toc485120467)

[SNAP-25 sensitivity analysis 8](#_Toc485120468)

[PSD-95 sensitivity analysis 10](#_Toc485120469)

[Septin 7 sensitivity analysis 11](#_Toc485120470)

[Gene Expression 12](#_Toc485120471)

[Immunohistochemical results 17](#_Toc485120472)

## ELISA results

Supplementary Table 1: Results from the ELISAs measuring protein levels in crude brain homogenates from the hippocampus and superior temporal gyrus. All results have been adjusted for total protein content and neuronal content. The ANOVA for PSD95 included postmortem delay as a co-variate.

| Brain Area | Variable Name | ε33  (n=57) | | ε34  (n=22) | | ε44  (n=5) | | ε32  (n=15) | | ε22  (n=1) | | Statistical Evidence (ANOVA or Kruskal Wallis) |
| --- | --- | --- | --- | --- | --- | --- | --- | --- | --- | --- | --- | --- |
|  |  | mean | SD | mean | SD | mean | SD | mean | SD | mean | SD |  |
| Hippocampus | Drebrin (ng/µl) | 5.208 | 2.698 | 5.846 | 3.067 | 7.444 | 5.578 | 8.6056 | 1.864 | 5.582 |  | p=0.011* |
| STG | Drebrin (ng/µl) | 6.070 | 3.901 | 6.080 | 3.830 | 6.001 | 3.467 | 6.567 | 4.52 | 6.406 |  | p=0.989 |
| Hippocampus | PSD-95 (ng/µl) | 0.605 | 0.628 | 0.647 | 0.849 | 0.746 | 0.726 | 1.208 | 1.565 | 0.237 |  | p=0.275 |
| STG | PSD-95 (ng/µl) | 0.580 | 0.587 | 0.589 | 0.413 | 0.682 | 0.438 | 0.611 | 0.598 | 0.308 |  | p=0.941 |
| Hippocampus | Septin-7 (ng/µl) | 71.590 | 50.031 | 70.293 | 66.153 | 79.281 | 50.721 | 96.091 | 38.949 | 21.706 |  | p=0.148 |
| STG | Septin-7 (ng/µl) | 60.016 | 48.600 | 63.319 | 48.910 | 63.056 | 28.445 | 68.466 | 41.645 | 37.875 |  | p=0.832 |
| Hippocampus | SNAP-25 (ng/ml) | 30.613 | 15.416 | 31.611 | 20.847 | 44.502 | 28.344 | 45.303 | 20.116 | 11.468 |  | p=0.056 |
| STG | SNAP-25 (ng/ml) | 34.73 | 24.809 | 32.149 | 20.277 | 28.738 | 25.866 | 29.619 | 15.451 | 17.654 |  | p=0.559 |
| Hippocampus | Synaptophysin (ng/µl) | 2.160 | 2.046 | 2.426 | 2.284 | 1.261 | 0.999 | 1.846 | 1.181 | 3.035 |  | p=0.702 |
| STG | Synaptophysin (ng/µl) | 2.579 | 1.725 | 2.650 | 2.404 | 3.190 | 1.947 | 3.433 | 2.842 | 2.436 |  | p=0.589 |
| Hippocampus | Z score post-synaptic  (arbitrary units) | 73.256 | 51.919 | 72.638 | 69.073 | 83.324 | 56.731 | 101.757 | 39.753 | 23.378 |  | Kruskal Wallis  Χ^2^ =11.696  p=0.020* |
| STG | Z score post-synaptic  (arbitrary units) | 63.384 | 51.898 | 65.971 | 51.938 | 65.722 | 31.163 | 70.77 | 42.684 | 40.571 |  | Kruskal Wallis  Χ^2^ =0.788  p=0.940 |
| Hippocampus | Z score pre-synaptic  (arbitrary units) | 29.900 | 16.311 | 30.879 | 20.797 | 42.890 | 28.662 | 44.276 | 20.512 | 11.630 |  | Kruskal Wallis  Χ^2^ =2.667  p=0.615 |
| STG | Z score pre-synaptic  (arbitrary units) | 34.514 | 25.519 | 32.004 | 21.906 | 29.133 | 27.282 | 30.257 | 16.246 | 17.295 |  | Kruskal Wallis  Χ^2^ =0.752  p=0.945 |

## The effect of postmortem delay

Supplementary Table 2: Pearson correlation between variables.

Significance is marked by a star if p<0.05

|  | Age at death (y) | Postmortem delay (h) |
| --- | --- | --- |
| Postmortem delay (h) | -0.3163* | 1 |
| Neuron specific enolase | 0.0322 | -0.2106* |
| Unadjusted drebrin | 0.1796* | -0.1997* |
| Adjusted drebrin | 0.0852 | 0.0520 |
| Unadjusted SNAP-25 | 0.2604* | -0.2392* |
| Adjusted SNAP-25 | 0.1853* | -0.0218 |
| Unadjusted PSD-95 | 0.118 | -0.2743* |
| Adjusted PSD-95 | 0.0434 | -0.0797 |
| Unadjusted synaptophysin | 0.2529* | -0.2834* |
| Adjusted synaptophysin | 0.118 | -0.0647 |
| Unadjusted septin 7 | 0.2550* | -0.3211* |
| Adjusted septin 7 | 0.1670* | -0.139 |

As shown in Supplementary Table 2, there was evidence to support several variables being correlated with age at death or postmortem delay. Age and gender were included in all regressions as co-variates, as is standard practice in genetic analyses. Although the vast majority of the proteins assayed in this project had been shown to be stable postmortem for up to 72 h, postmortem delay was included as a co-variate in all regressions as many of the samples had postmortem delays significantly longer than 72 h.

## The effect of Braak Stage

As shown in Supplementary Figure 1 the observed increase in drebrin in ε32 group in the hippocampus was seen regardless of Braak stage.


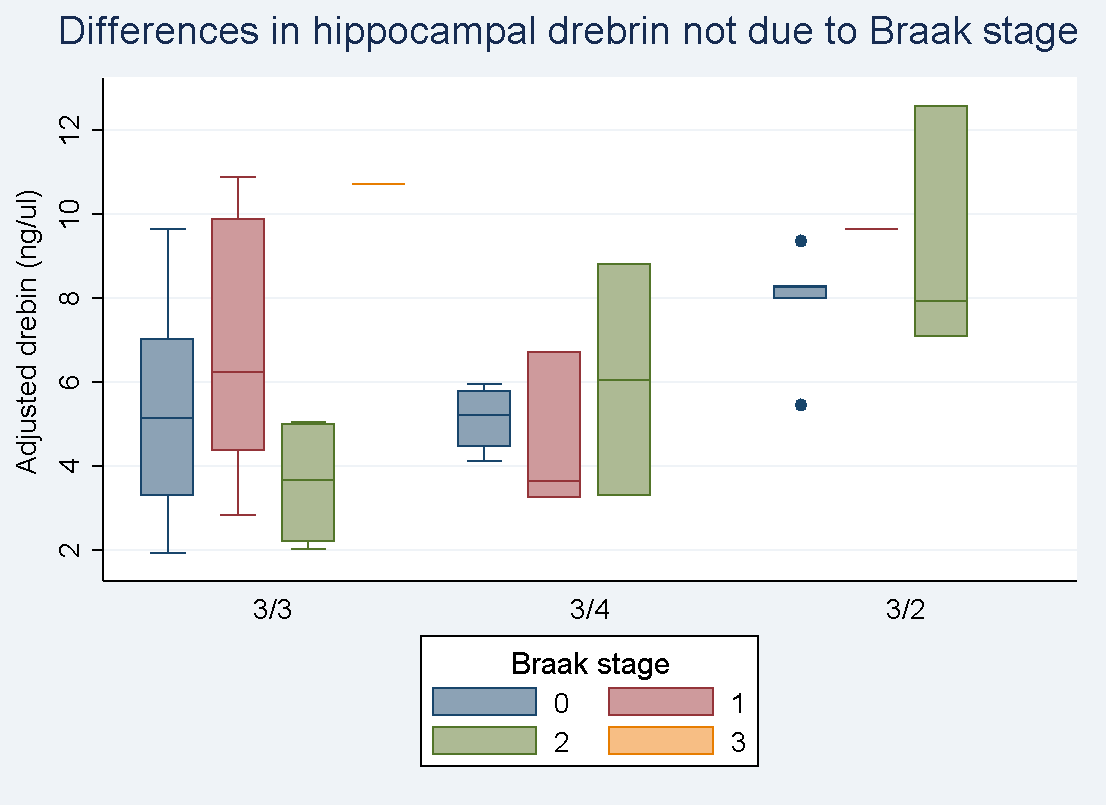


Supplementary Figure 1: Drebrin in the hippocampus in relation to Braak stage

**Postmortem stability of septin 7**

As can be seen from Supplementary Figure 2, there was no biologically relevant decrease in septin 7 at up to 72hrs of storage either at room temperature (ρ = -0.07143, p = 0.9063) or at 4ºC (ρ = -0.8000, p=0.3333).


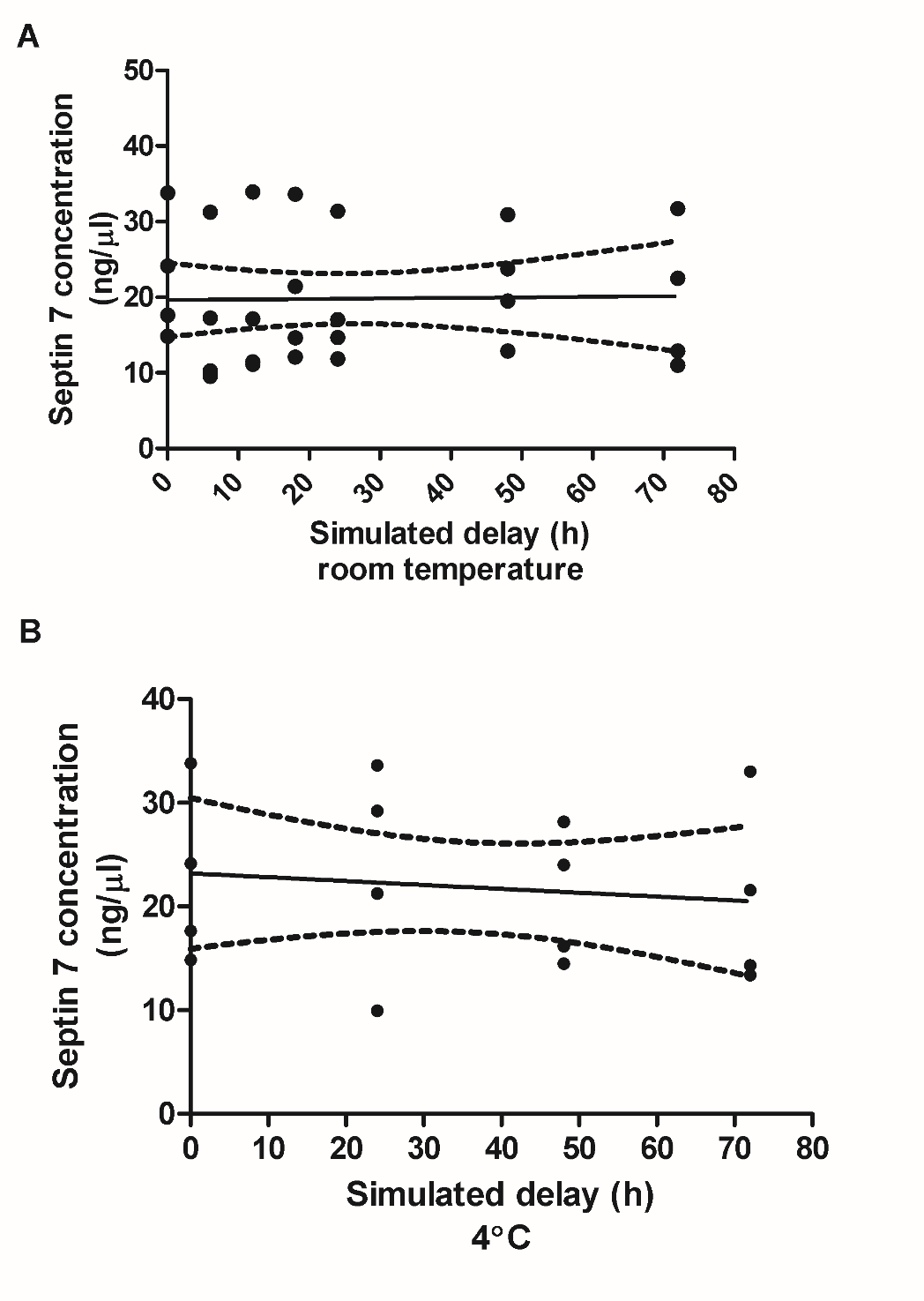


Supplementary Figure 2: The effect of postmortem delay on septin 7 concentrations was simulated by storing tissue with a short postmortem delay at room temperature (A) or 4ºC (B) for various periods of time. There was no evidence of a decline in septin 7 concentrations at room temperature (Spearman’s ρ=0.07143, p=0.9063) or 4ºC (Spearman’s ρ=-0.8000, p=0.3333). Graphs show the best fit linear regression line and its 95% confidence intervals.

## Drebrin sensitivity analysis

**Hippocampus**

Supplementary Table 3: Results from the linear regression of log drebrin adjusted for total protein content and neuronal content. ε33 was the reference group.

|  | Co-efficient | 95% CI | p value |
| --- | --- | --- | --- |
| ε44 | 0.188 | -0.293 to 0.669 | 0.439 |
| ε34 | 0.129 | -0.136 to 0.394 | 0.336 |
| ε33 | 0 | 0 | N/A |
| ε32 | 0.614 | 0.285 to 0.943 | <0.001 * |
| ε22 | 0.203 | -0.836 to 1.241 | 0.699 |

Supplementary Table 4: In a sensitivity analysis, performed because some of the samples included had a postmortem delay of longer than 72 h the finding of increased drebrin in the ε32 group was essentially unchanged. In this instance, the regression of log drebrin included age at death and postmortem delay as co-variates.

|  | Co-efficient | 95% CI | p value |
| --- | --- | --- | --- |
| ε44 | 0.286 | -0.209 to 0.781 | 0.253 |
| ε34 | 0.149 | -0.116 to 0.414 | 0.267 |
| ε33 | 0 | 0 | N/A |
| ε32 | 0.426 | 0.0707 to 0.780 | 0.019 * |
| ε22 | 0.2123 | -0.827 to 1.253 | 0.685 |

**STG**

Supplementary Table 5: Results of the regressions of log drebrin adjusted for NSE in the superior temporal gyrus. All regressions included age at death, postmortem delay, gender and brain bank center as co-variates with ε33 as the reference group.

|  | Co-efficient | 95% CI | p value |
| --- | --- | --- | --- |
| ε4 (compared to non ε4) | -0.018 | -0.296 to 0.260 | 0.897 |
| ε44 | 0.109 | -0.484 to 0.702 | 0.715 |
| ε34 | -0.023 | -0.334 to 0.287 | 0.882 |
| ε33 | 0 | 0 | N/A |
| ε32 | 0.102 | -0.283 to 0.486 | 0.600 |
| ε22 | 0.209 | -1.039 to 1.4574 | 0.740 |

Supplementary Table 6: Sensitivity analysis for drebrin in the STG. All samples with a postmortem delay of >72 h were removed from the analysis.

|  | Co-efficient | 95% CI | p value |
| --- | --- | --- | --- |
| ε44 | 0.357 | -0.283 to 0.998 | 0.270 |
| ε34 | -0.059 | -0.383 to 0.265 | 0.717 |
| ε33 | 0 | 0 | N/A |
| ε32 | 0.016 | -0.394 to 0.427 | 0.937 |

## Synaptophysin Sensitivity Analysis

**Hippocampus**

Supplementary Table 7: Results of the regressions of log synaptophysin adjusted for NSE in the hippocampus.

All regressions included age at death, postmortem delay, gender and brain bank center as co-variates with ε33 as the reference group.

|  | Co-efficient | 95% CI | p value |
| --- | --- | --- | --- |
| ε44 | -0.324 | -1.1842 to 0.536 | 0.455 |
| ε34 | 0.093 | -.367 to 0 .553 | 0.689 |
| ε33 | 0 | 0 | N/A |
| ε32 | -0.198 | -0.815 to 0.419 | 0.525 |
| ε22 | 0.782 | -1.025 to 2.589 | 0.392 |

Supplementary Table 8: The same analysis re-run for the hippocampal samples without the individuals with a postmortem delay of >72 h.

|  | Co-efficient | 95% CI | p value |
| --- | --- | --- | --- |
| ε44 | -0.330 | -1.331 to 0.671 | 0.513 |
| ε34 | 0.143 | -0.375 to 0.661 | 0.584 |
| ε33 | 0 | 0 | N/A |
| ε32 | -0.180 | -0.907 to 0.548 | 0.624 |

**STG**

Supplementary Table 9: Results of the regressions of log synaptophysin adjusted for NSE in the superior temporal gyrus.

All regressions included age at death, postmortem delay, gender and brain bank center as co-variates with ε33 as the reference group.

|  | Co-efficient | 95% CI | p value |
| --- | --- | --- | --- |
| ε44 | 0.414 | -0.340 to 1.168 | 0.278 |
| ε34 | -0.121 | -0.515 to 0.274 | 0.545 |
| ε33 | 0 | 0 | N/A |
| ε32 | 0.048 | -0.440 to 0.537 | 0.844 |
| ε22 | 0.183 | -1.404 to 1.771 | 0.819 |

Supplementary Table 10: The analysis re-run for the STG excluding samples with a postmortem delay of >72 h.

|  | Co-efficient | 95% CI | p value |
| --- | --- | --- | --- |
| ε44 | 0.593 | -0.265 to 1.452 | 0.173 |
| ε34 | -0.197 | -0.631 to 0.237 | 0.369 |
| ε33 | 0 | 0 | N/A |
| ε32 | 0.809 | -0.469 to 0.631 | 0.770 |

## SNAP-25 sensitivity analysis

**Hippocampus**

Supplementary Table 11: Results of the regressions of log SNAP-25 adjusted for NSE in the hippocampus. All regressions included age at death, postmortem delay, gender and brain bank center as co-variates with ε33 as the reference group.

|  | Co-efficient | 95% CI | p value |
| --- | --- | --- | --- |
| ε4 (compared to non ε4) | -0.075 | -0.182 to 0.332 | 0.563 |
| ε44 | 0.428 | -0.085 to 0.942 | 0.101 |
| ε34 | 0.011 | -0.269 to 0.290 | 0.940 |
| ε33 | 0 | 0 | N/A |
| ε32 | 0.189 | -0.180 to 0.558 | 0.310 |
| ε22 | -0.889 | -0.178 to 0.558 | 0.105 |

Supplementary Table 12: Sensitivity analysis for SNAP-25 in the hippocampus. All samples with a postmortem delay of >72 h have been removed from the analysis.

|  | Co-efficient | 95% CI | p value |
| --- | --- | --- | --- |
| ε44 | 0.159 | -0.375 to 0.694 | 0.554 |
| ε34 | -0.017 | -0.300 to 0.266 | 0.906 |
| ε33 | 0 | 0 | N/A |
| ε32 | 0.225 | -0.165 to 0.615 | 0.253 |

**STG**

Supplementary Table 13: Results of the regressions of log SNAP-25 adjusted for NSE in the superior temporal gyrus. All regressions included age at death, postmortem delay, gender and brain bank center as co-variates with ε33 as the reference group.

|  | Co-efficient | 95% CI | p value |
| --- | --- | --- | --- |
| ε4 (compared to non ε4) | -0.091 | -0.402 to 0.220 | 0.563 |
| ε44 | -0.356 | -1.018 to 0.306 | 0.289 |
| ε34 | -0.062 | -0.409 to 0.284 | 0.723 |
| ε33 | 0 | 0 | N/A |
| ε32 | -0.118 | -0.547 to 0.311 | 0.585 |
| ε22 | -0.400 | -1.794 to 0.993 | 0.569 |

Supplementary Table 14: Sensitivity analysis for SNAP-25 in the STG. All samples with a postmortem delay of >72 h have been removed from the analysis.

|  | Co-efficient | 95% CI | p value |
| --- | --- | --- | --- |
| ε44 | -0.286 | -1.043 to 0.473 | 0.455 |
| ε34 | -0.084 | -0.468 to 0.299 | 0.662 |
| ε33 | 0 | 0 | N/A |
| ε32 | -0.069 | -0.555 to 0.416 | 0.776 |

## PSD-95 sensitivity analysis

**Hippocampus**

Supplementary Table 15: Results of the regressions of log PSD-95 adjusted for NSE in the hippocampus. All regressions included age at death, postmortem delay, gender and brain bank center as co-variates with ε33 as the reference group.

|  | Co-efficient | 95% CI | p value |
| --- | --- | --- | --- |
| ε4 (compared to non ε4) | 0.041 | -0.319 to 0.401 | 0.822 |
| ε44 | 0.147 | -0.593 to 0.887 | 0.694 |
| ε34 | 0.093 | -0.303 to 0.489 | 0.643 |
| ε33 | 0 | 0 | N/A |
| ε32 | 0.475 | -0.056 to 1.006 | 0.079 |
| ε22 | -0.545 | -2.100 to 1.011 | 0.488 |

Supplementary Table 16: Sensitivity analysis for PSD-95 in the hippocampus. All samples with a postmortem delay of >72 h have been removed from the analysis. As can be seen there is weak evidence of a rise in PSD-95 in the ε32 group.

|  | Co-efficient | 95% CI | p value |
| --- | --- | --- | --- |
| ε44 | 0.042 | -0.802 to 0.886 | 0.921 |
| ε34 | 0.059 | -0.378 to 0.496 | 0.789 |
| ε33 | 0 | 0 | N/A |
| ε32 | 0.597 | -0.156 to 1.210 | 0.056 |

**STG**

Supplementary Table 17: Results of the regressions of log PSD-95 adjusted for NSE in the superior temporal gyrus. All regressions included age at death, postmortem delay, gender and brain bank center as co-variates with ε33 as the reference group.

|  | Co-efficient | 95% CI | p value |
| --- | --- | --- | --- |
| ε4 (compared to non ε4) | 0.098 | -0.277 to 0.473 | 0.603 |
| ε44 | 0.472 | -0.322 to 1.265 | 0.241 |
| ε34 | 0.033 | -0.384 to 0.451 | 0.875 |
| ε33 | 0 | 0 | N/A |
| ε32 | 0.120 | -0.395 to 0.635 | 0.645 |
| ε22 | -0.254 | -1.925 to 1.417 | 0.763 |

Supplementary Table 18: Sensitivity analysis for PSD-95 in the STG. All samples with a postmortem delay of >72 h have been removed from the analysis.

|  | Co-efficient | 95% CI | p value |
| --- | --- | --- | --- |
| ε44 | 0.742 | -0.131 to 1.614 | 0.094 |
| ε34 | 0.029 | -0.414 to 0.473 | 0.895 |
| ε33 | 0 | 0 | N/A |
| ε32 | 0.414 | -1.450 to 0.973 | 0.144 |

## Septin 7 sensitivity analysis

**Hippocampus**

Supplementary Table 19: Results of the regressions of log septin 7 adjusted for NSE in the hippocampus. All regressions included age at death, postmortem delay, gender and brain bank center as co-variates with ε33 as the reference group.

|  | Co-efficient | 95% CI | p value |
| --- | --- | --- | --- |
| ε4 (compared to non ε4) | 0.024 | -0.270 to 0.317 | 0.872 |
| ε44 | 0.292 | -0.310 to 0.894 | 0.337 |
| ε34 | -0.018 | -0.340 to 0.304 | 0.911 |
| ε33 | 0 | 0 | N/A |
| ε32 | 0.202 | -0.230 to 0.633 | 0.355 |
| ε22 | -0.903 | -2.168 to 0.362 | 0.159 |

Supplementary Table 20: Sensitivity analysis for septin7 in the hippocampus. All samples with a postmortem delay of >72 h have been removed from the analysis.

|  | Co-efficient | 95% CI | p value |
| --- | --- | --- | --- |
| ε44 | 0.090 | -0.569 to 0.749 | 0.787 |
| ε34 | -0.046 | -0.387 to 0.295 | 0.788 |
| ε33 | 0 | 0 | N/A |
| ε32 | 0.245 | -0.234 to 0.724 | 0.312 |

**STG**

Supplementary Table 21: Results of the regressions of log septin 7 adjusted for NSE in the superior temporal gyrus. All regressions included age at death, postmortem delay, gender and brain bank center as co-variates with ε33 as the reference group.

|  | Co-efficient | 95% CI | p value |
| --- | --- | --- | --- |
| ε4 (compared to non ε4) | 0.089 | -0.235 to 0.413 | 0.588 |
| ε44 | .3803609 | -0.308 to 1.068 | 0.275 |
| ε34 | .0478127 | -0.312 to 0.408 | 0.792 |
| ε33 | 0 | 0 | N/A |
| ε32 | 0.127 | -0.336 to 0.591 | 0.586 |
| ε22 | 0.082 | -1.368 to 1.532 | 0.911 |

Supplementary Table 22: Sensitivity analysis for septin7 in the STG. All samples with a postmortem delay of >72 h have been removed from the analysis.

|  | Co-efficient | 95% CI | p value |
| --- | --- | --- | --- |
| ε44 | 0.863 | 0.143 to 1.582 | 0.391 |
| ε34 | 0.158 | -0.206 to 0.521 | 0.391 |
| ε33 | 0 | 0 | N/A |
| ε32 | 0.171 | -0.289 to 0.632 | 0.461 |

## Gene Expression

To maximize study power the initial analysis was performed as ε4 versus non ε4. To further explore the findings a follow-up analysis using all of the *APOE* genotype groups was performed. This is limited by very low study power. This analysis did not suggest any per genotype differences in the superior temporal gyrus. There was limited evidence of

Supplementary Table 23: Summary RT-PCR results for the Hippocampal Samples.

The ANOVAs for all analyses where the calibrator was ENO2 included age at death as a co-variate and, where GAPDH was the calibrator, postmortem delay. Due to non-normal distributions, the majority of the analyses used the Kruskal-Wallis test.

|  |  | ϵ22  (n=1) | | | ϵ32  (n=12) | | | ϵ33  (n=52) | | | ϵ34  (n=20) | | | ϵ44  (n=5) | | | Statistical evidence for a between group difference  (ANOVA or Kruskal Wallis) |
| --- | --- | --- | --- | --- | --- | --- | --- | --- | --- | --- | --- | --- | --- | --- | --- | --- | --- |
|  | Vs | Geo mean | Median | SD | Geo mean | Median | SD | Geo mean | Median | SD | Geo mean | Median | SD | Geo mean | Median | SD |  |
| *DBN1* | *ENO2* | 0.960 | 0.960 |  | 0.799 | 0.946 | 0.308 | 1.041 | 1.057 | 1.405 | 1.068 | 0.991 | 0.711 | 0.899 | 1.039 | 0.626 | p=0.745 |
|  | *GAPDH* | 0.850 | 0.850 |  | 0.824 | 0.730 | 0.520 | 1.063 | 1.051 | 0.890 | 1.031 | 0.849 | 2.102 | 1.323 | 1.223 | 0.711 | Χ^2^=3.995, p=0.407 |
|  | *MAP2* | 0.720 | 0.720 |  | 0.930 | 1.138 | 0.314 | 0.998 | 0.998 | 0.949 | 1.099 | 1.060 | 2.022 | 0.929 | 1.050 | 0.341 | Χ^2^=0.711, p=0.950 |
| *DLG4* | *ENO2* | 0.930 | 0.930 |  | 0.853 | 1.004 | 0.664 | 0.919 | 0.963 | 0.972 | 1.420 | 1.479 | 1.590 | 0.980 | 1.023 | 1.001 | p=0.199 |
|  | *GAPDH* | 0.840 | 0.840 |  | 0.898 | 0.872 | 0.308 | 0.962 | 1.010 | 0.741 | 1.253 | 1.214 | 0.757 | 1.474 | 1.384 | 0.788 | p=0.126 |
|  | *MAP2* | 0.690 | 0.690 |  | 0.995 | 1.216 | 0.408 | 0.878 | 1.145 | 0.571 | 1.465 | 1.416 | 2.851 | 1.015 | 1.221 | 0.475 | Χ^2^=8.401, p=0.078 |
| *ENO2* | *GAPDH* | 0.880 | .880 |  | 1.033 | 0.934 | 0.769 | 1.02 | 1.022 | 0.690 | 0.837 | 0.885 | 0.544 | 1.475 | 1.594 | 0.967 | Χ^2^=5.25, p=0.263 |
|  | *MAP2* | 0.740 | 0.740 |  | 1.164 | 1.246 | 0.395 | 0.959 | 0.958 | 0.477 | 1.028 | 1.009 | 1.389 | 1.033 | 0.851 | 1.126 | p=0.761 |
| *MAP2* | *GAPDH* |  |  |  |  |  |  |  |  |  |  |  |  |  |  |  | Χ^2^=6.364, p=0.174 |
| *SEPT7* | *ENO2* | 0.730 | 0.730 |  | 0.810 | 0.810 | 0.685 | 1.045 | 1.018 | 2.797 | 1.113 | 1.097 | 3.014 | 0.778 | 0802 | 0.602 | Χ^2^=2.030, p=0.719 |
|  | *GAPDH* | 0.640 | 0.640 |  | 0.820 | 0.849 | 0.718 | 1.060 | 1.023 | 3.392 | 1.079 | 0.941 | 3.56 | 1.124 | 1.058 | 0.605 | Χ^2^=1.292, p=0.863 |
|  | *MAP2* | 0.550 | 0.550 |  | 0.948 | 0.992 | 0.555 | 1.007 | 0.845 | 2.455 | 1.121 | 1.037 | 2.800 | 0.808 | 0.574 | 0.613 | Χ^2^=2.093, p=0.719 |
| *SNAP25* | *ENO2* | 0.790 | 0.790 |  | 0.684 | 0.716 | 0.189 | 1.092 | 0.997 | 1.256 | 1.058 | 1.080 | 0.767 | 0.867 | 1.128 | 0.434 | Χ^2^=10.048, p=0.040 * |
|  | *GAPDH* | 0.730 | 0.730 |  | 0.736 | 0.620 | 0.543 | 1.133 | 1.018 | 1.052 | 0.796 | 0.676 | 2.502 | 1.334 | 1.286 | 0.654 | Χ^2^=12.676, p=0.013 * |
|  | *MAP2* | 0.590 | 0.590 |  | 0.803 | 0.750 | 0.322 | 1.040 | 1.016 | 0.699 | 1.098 | 0.967 | 2.249 | 0.904 | 0.969 | 0.152 | Χ^2^=5.655, p=0.227 |
| *SYP* | *ENO2* | 1.470 | 1.470 |  | 0.798 | 0.874 | 0.289 | 1.091 | 1.044 | 0.822 | 0.925 | 0.906 | 0.478 | 0.850 | 1.029 | 0.456 | p=0.180 |
|  | *GAPDH* | 1.310 | 1.310 |  | 0.826 | 0.792 | 0.755 | 1.123 | 1.043 | 1.198 | 0.875 | 0.639 | 1.635 | 1.257 | 1.024 | 0.726 | Χ^2^=5.959, p=0.202 |
|  | *MAP2* | 1.100 | 1.100 |  | 0.928 | 0.958 | 0.396 | 1.046 | 1.007 | 0.746 | 0.952 | 0.856 | 0.897 | 0.878 | 0.897 | 0.189 | p=0.891 |

Supplementary Table 24: Summary RT-PCR results for the Superior Temporal Gyrus Samples.

The ANOVAs for all analyses where the calibrator was ENO2 included age at death as a co-variate and, where GAPDH was the calibrator, postmortem delay. Due to non-normal distributions the majority of the analyses used Kruskal-Wallis test.

|  |  | ϵ22  (n=1) | | | ϵ32  (n=13) | | | ϵ33  (n=57) | | | ϵ34  (n=21) | | | ϵ44  (n=5) | | | | Statistical evidence for a between group difference  (ANOVA or Kruskal Wallis) | |
| --- | --- | --- | --- | --- | --- | --- | --- | --- | --- | --- | --- | --- | --- | --- | --- | --- | --- | --- | --- |
|  | Vs | Geo mean | Median | SD | Geo mean | Median | SD | Geo mean | Median | SD | Geo mean | Median | SD | Geo mean | Median | | SD |  |  |
| *DBN1* | *ENO2* | 1.000 | 1.000 |  | 0.660 | 0.704 | 2.686 | 1.163 | 0.867 | 5.830 | 0.970 | 0.814 | 0.884 | 0.680 | 0.571 | 0.432 | | Χ^2^=2.899, p=0.575 |  |
|  | *GAPDH* | 0.940 | 0.940 |  | 0.711 | 0.610 | 0.976 | 1.172 | 0.910 | 4.365 | 0.956 | 0.849 | 1.061 | 0.847 | 0.918 | 0.246 | | Χ^2^=3.563, p=0.468 |  |
|  | *MAP2* | 0.620 | 0.620 |  | 0.556 | 0.638 | 0.682 | 1.311 | 1.100 | 6.937 | 0.830 | 0.666 | 0.885 | 0.665 | 0.685 | 0.165 | | Χ^2^=9.426, p=0.051 |  |
| *DLG4* | *ENO2* | 2.030 | 2.030 |  | 0.638 | 0.920 | 0.522 | 1.005 | 0.891 | 0.805 | 1.199 | 1.330 | 1.032 | 1.262 | 1.166 | 1.040 | | Χ^2^=4.481, p=0.345 |  |
|  | *GAPDH* | 1.860 | 1.860 |  | 0.671 | 0.984 | 0.626 | 0.996 | 1.041 | 0.519 | 1.152 | 1.136 | 0.660 | 1.591 | 1.547 | 0.498 | | Χ^2^=7.961, p=0.093 |  |
|  | *MAP2* | 1.280 | 1.280 |  | 0.547 | 1.105 | 0.631 | 1.086 | 1.231 | 0.536 | 1.007 | 1.198 | 0.578 | 1.468 | 1.469 | 0.329 | | Χ^2^=2.653, p=0.617 |  |
| *ENO2* | *GAPDH* | 0.910 | 0.910 |  | 1.046 | 0.884 | 11.476 | 1.009 | 0.970 | 1.270 | 0.974 | 0.929 | 0.522 | 1.210 | 1.043 | 0.633 | | Χ^2^=0.763, p=0.943 |  |
|  | *MAP2* | 0.620 | 0.620 |  | 0.846 | 0.994 | 0.576 | 1.124 | 1.005 | 0.906 | 0.820 | 0.873 | 0.348 | 1.099 | 0.916 | 0.794 | | Χ^2^=4.751, p=0.314 |  |
| *MAP2* | *GAPDH* |  |  |  |  |  |  |  |  |  |  |  |  |  |  |  | | Χ^2^=3.423, p=0.490 |  |
| *SEPT7* | *ENO2* | 0.910 | 0.910 |  | 1.025 | 0.695 | 4.601 | 0.892 | 0.849 | 1.523 | 1.190 | 0.923 | 1.409 | 1.656 | 1.035 | 9.804 | | Χ^2^=3.590, p=0.464 |  |
|  | *GAPDH* | 0.840 | 0.840 |  | 1.093 | 0.609 | 16.224 | 0.858 | 0.776 | 0.835 | 1.180 | 1.034 | 2.077 | 2.040 | 1.243 | 7.517 | | Χ^2^=5.927, p=0.205 |  |
|  | *MAP2* | 0.560 | 0.560 |  | 0.854 | 0.784 | 0.623 | 0.976 | 0.866 | 3.710 | 1.041 | 0.831 | 1.168 | 1.953 | 1.073 | 6.415 | | Χ^2^=3.197, p=0.525 |  |
| *SNAP25* | *ENO2* | 1.430 | 1.430 |  | 0.650 | 0.827 | 0.848 | 1.029 | 0.966 | 1.946 | 1.248 | 1.113 | 0.912 | 0.792 | 0.978 | 0.453 | | Χ^2^=4.525, p=0.340 |  |
|  | *GAPDH* | 1.340 | 1.340 |  | 0.701 | 0.798 | 0.322 | 1.014 | 0.847 | 1.342 | 1.254 | 1.093 | 1.574 | 0.988 | 1.052 | 0.421 | | Χ^2^=3.436, p=0.488 |  |
|  | *MAP2* | 0.970 | 0.970 |  | 0.599 | 0.860 | 0.473 | 1.238 | 1.109 | 2.671 | 0.786 | 0.625 | 1.022 | 0.947 | 0.976 | 0.261 | | Χ^2^=7.882, p=0.096 |  |
| *SYP* | *ENO2* | 1.060 | 1.060 |  | 0.790 | 0.950 | 0.302 | 0.955 | 0.991 | 0.707 | 1.281 | 1.267 | 0.821 | 1.062 | 1.264 | 0.459 | | p= 0.086 |  |
|  | *GAPDH* | 0.980 | 0.980 |  | 0.837 | 0.810 | 13.439 | 0.930 | 0.776 | 1.174 | 1.262 | 1.085 | 1.351 | 1.301 | 1.299 | 0.490 | | Χ^2^=5.573, p=0.233 |  |
|  | *MAP2* | 0.660 | 0.660 |  | 0.664 | 0.878 | 0.424 | 1.065 | 1.064 | 1.579 | 1.057 | 1.056 | 1.027 | 1.160 | 1.288 | 0.362 | | Χ^2^=6.043, p=0.196 |  |

Supplementary Table 25: Hippocampal RT-PCR results when analyzed per possession of an ϵ4 allele.

The ANOVAs for all analyses where the calibrator was ENO2 included age at death as a co-variate and, where GAPDH was the calibrator, postmortem delay. *p value <0.05

|  |  | **Non-ε4** | | | **ε4** | | | Statistical evidence for a between group difference  (ANOVA or Kruskal Wallis) |  |
| --- | --- | --- | --- | --- | --- | --- | --- | --- | --- |
|  | Vs | geometric mean | median | SD | geometric mean | median | SD |  |  |
| *DBN1* | *ENO2* | 0.990 | 0.960 | 1.278 | 1.029 | 0.998 | 0.683 | p=0.855 | |
|  | *GAPDH* | 1.010 | 0.937 | 0.834 | 1.089 | 0.956 | 1.873 | Χ^2^=0.107, p=0.744 | |
|  | *MAP2* | 0.980 | 0.998 | 0.864 | 1.059 | 1.050 | 1.799 | Χ^2^=0.087, p=0.769 | |
| *DLG4* | *ENO2* | 0.906 | 0.961 | 0.912 | 1.310 | 1.478 | 1.477 | p=0.047* | |
|  | *GAPDH* | 0.947 | 0.944 | 0.676 | 1.298 | 1.284 | 0.752 | p=0.021* | |
|  | *MAP2* | 0.896 | 1.146 | 0.538 | 1.352 | 1.369 | 2.548 | Χ^2^=6.609, p=0.010* | |
| *ENO2* | *GAPDH* | 1.020 | 1.012 | 0.695 | 0.946 | 0.939 | 0.702 | Χ^2^=0.535, p=0.465 | |
|  | *MAP2* | 0.990 | 0.984 | 0.463 | 1.029 | 0.877 | 1.312 | p=0.748 | |
| *SEPT7* | *ENO2* | 0.991 | 0.951 | 2.524 | 1.026 | 0.945 | 2.676 | Χ^2^=0.005, p=0.946 | |
|  | *GAPDH* | 1.002 | 0.900 | 3.056 | 1.090 | 0.967 | 3.136 | Χ^2^=0.005, p=0.943 | |
|  | *MAP2* | 0.987 | 0.852 | 2.213 | 1.040 | 0.969 | 2.494 | Χ^2^=0.110, p=0.740 | |
| *SNAP25* | *ENO2* | 0.995 | 0.915 | 1.150 | 1.013 | 1.106 | 0.705 | Χ^2^=0.750, p=0.386 | |
|  | *GAPDH* | 1.037 | 0.976 | 0.987 | 0.890 | 0.840 | 2.217 | Χ^2^=1.960, p=0.162 | |
|  | *MAP2* | 0.982 | 0.914 | 0.652 | 1.053 | 0.969 | 1.997 | Χ^2^=0.063, p=0.802 | |
| *SYP* | *ENO2* | 1.035 | 0.996 | 0.763 | 0.908 | 0.945 | 0.463 | p=0.218 | |
|  | *GAPDH* | 1.063 | 0.965 | 1.126 | 0.947 | 0.789 | 1.470 | Χ^2^=1.402, p=0.237 | |
|  | *MAP2* | 1.024 | 1.005 | 0.691 | 0.935 | 0.875 | 0.798 | p=0.474 | |

Supplementary Table 26: Superior Temporal Gyrus RT-PCR results when analyzed per possession of an ε4 allele

|  |  | **Non-ε4** | | | **ε4** | | | Statistical evidence for a between group difference  (ANOVA or Kruskal Wallis) |
| --- | --- | --- | --- | --- | --- | --- | --- | --- |
|  | Vs | geometric mean | median | SD | geometric mean | median | SD |  |
| *DBN1* | *ENO2* | 1.048 | 0.838 | 5.358 | 0.901 | 0.812 | 0.826 | Χ^2^ =0.117, p=0.732 |
|  | *GAPDH* | 1.066 | 0.840 | 3.962 | 0.932 | 0.905 | 0.959 | Χ^2^=0.004, p=0.834 |
|  | *MAP2* | 1.112 | 1.024 | 6.287 | 0.794 | 0.685 | 0.806 | Χ^2^=3.544, p=0.060 |
| *DLG4* | *ENO2* | 0.932 | 0.895 | 0.768 | 1.210 | 1.291 | 1.010 | Χ^2^=1.933, p=0.164 |
|  | *GAPDH* | 0.932 | 1.046 | 0.542 | 1.219 | 1.157 | 0.642 | Χ^2^=2.261, p=0.133 |
|  | *MAP2* | 0.955 | 1.203 | 0.553 | 1.072 | 1.237 | 0.553 | Χ^2^=0.087, p=0.769 |
| *ENO2* | *GAPDH* | 1.014 | 0.941 | 5.029 | 1.017 | 0.992 | 0.539 | Χ^2^=0.102, p=0.749 |
|  | *MAP2* | 1.057 | 0.999 | 0.856 | 0.869 | 0.898 | 0.472 | Χ^2^=2.126, p=0.145 |
| *SEPT7* | *ENO2* | 0.914 | 0.839 | 2.362 | 1.271 | 0.949 | 4.464 | Χ^2^=3.222, p=0.073 |
|  | *GAPDH* | 0.896 | 0.775 | 6.945 | 1.317 | 1.151 | 3.770 | Χ^2^=4.515, p=0.034 * |
|  | *MAP2* | 0.945 | 0.856 | 3.35 | 1.152 | 0.979 | 2.728 | Χ^2^=0.975, p=0.323 |
| *SNAP25* | *ENO2* | 0.952 | 0.959 | 1.786 | 1.140 | 1.038 | 0.866 | Χ^2^=1.292, p=0.256 |
|  | *GAPDH* | 0.952 | 0.845 | 1.233 | 1.196 | 1.052 | 1.436 | Χ^2^=1.572, p=0.210 |
|  | *MAP2* | 1.083 | 1.071 | 2.426 | 0.815 | 0.819 | 0.921 | Χ^2^=4.327, p=0.038 * |
| *SYP* | *ENO2* | 0.925 | 0.982 | 0.652 | 1.234 | 1.264 | 0.764 | p=0.018 * |
|  | *GAPDH* | 0.913 | 0.776 | 5.780 | 1.270 | 1.101 | 1.224 | Χ^2^=5.040, p=0.025 * |
|  | *MAP2* | 0.972 | 0.996 | 1.443 | 1.076 | 1.071 | 0.930 | Χ^2^=0.469, p=0.493 |

## Immunohistochemical results

Supplementary Table 27: Results from the immunohistochemical field fraction experiment. Field fractions were assessed using random fields from CA1,2 and 3.

|  | **ε32 (n=14)**  **mean (SD)** | **ε33 (n=14)**  **mean (SD)** | **ε34 (n=13)**  **mean (SD)** | **Statistical evidence (ANOVA/Kruskal Wallis)** |
| --- | --- | --- | --- | --- |
| Age (y) | 60.4 (11.7) | 60.5 (12.5) | 61.3 (10.5) | p=0.979 |
| Gender  *Male*  *Female* | 11  2 | 11  1 | 11  2 |  |
| Postmortem delay (h) | 50.5 (24.8) | 53.3 (22.3) | 52.5 (20.4) | p=0.951 |
| PSD95 mean field fraction (%) | 6.3 (2.9) | 4.1 (2.1) | 6.6 (3.3) | (Kruskal Wallis) p=0.066 |
| MAP2 mean field fraction (%) | 9.7 (3.2) | 11.6 (3.0) | 11.1 (2.7) | p=0.369 |
| Drebrin mean field fraction (%) | 5.4 (1.2) | 5.2 (0.8) | 5.4 (1.1) | p=0.884 |
